# Severe Pulmonary Hypertension Due to Adult-Onset Still’s Disease

**DOI:** 10.1177/2324709618757260

**Published:** 2018-02-13

**Authors:** Ankur Sinha, Ravikaran Patti, Paurush Ambesh, Chukwudi Obiagwu, Namrita Malhan, Kabu Chawla

**Affiliations:** 1 Maimonides Medical Center, Brooklyn, NY, USA; 2 Sri Aurobindo Institute of Medical Sciences, Indore, India

**Keywords:** pulmonary hypertension, adult-onset Still’s disease

## Abstract

A 29-year-old female with adult-onset Still’s disease (AOSD) presented with progressive shortness of breath both on rest and on exertion, increased abdominal girth, and swelling in both legs. She was on oral prednisone and was recently started on canakinumab (interleukin-1 antagonist) for joint pain and rash of AOSD. Echocardiogram showed severely dilated right ventricle, dilated pulmonary artery, moderately reduced right ventricular systolic function, but with normal left ventricular systolic function. Computed tomography with contrast ruled out pulmonary embolism. Blood tests ruled out other rheumatologic diseases. The patient was diagnosed with right-sided heart failure likely secondary to AOSD. Right heart catheterization was needed but could not be performed because of severely dilated pulmonary artery. The patient was transferred to a higher center for further management and possible cardiopulmonary transplant.

## Introduction

Adult-onset Still’s disease (AOSD) is a rare inflammatory autoimmune disease that is classically described by “Still’s triad” of fever, maculopapular rash, and arthritis, but it also has other atypical features like leukocytosis, elevated liver enzymes, and high serum ferritin levels.^1^ The etiology remains largely unknown.

Pulmonary arterial hypertension (PAH) is a disease characterized by progressive constriction of pulmonary arterioles, thereby causing an elevation in pulmonary arterial resistance and pressure. Although PAH has been reported with connective tissue disorders like systemic lupus erythematosus and systemic sclerosis, its association with AOSD is very rare.

## Case Report

A 29-year-old female with AOSD presented with shortness of breath at rest. Her symptoms had progressed over 3 months. She complained of increased abdominal girth with swelling in both her legs. She had a history of poorly controlled AOSD with frequent flares leading to joint pain and rash. On discussion with the patient’s primary rheumatologist, we gathered that the patient had met criteria for diagnosis of AOSD (Yamaguchi criteria). The major criteria met were intermittent arthralgia lasting more than 4 weeks and leukocytosis as high as 13 000/mm with 91% neutrophils without evidence of infection. The minor criteria satisfying the diagnosis of AOSD were cervical lymphadenopathy on presentation, abnormal liver function tests (raised alkaline phosphatase to 138 U/L, aspartate aminotransferase of 75 U/L, and alanine aminotransferase of 60 U/L), a negative antinuclear antibody, and a negative rheumatoid factor. She met 5 criteria of AOSD with 2 major and 3 minor criteria. Workup for other rheumatologic conditions including systemic lupus erythematosus, systemic sclerosis, and CREST (calcinosis, Raynaud phenomenon, esophageal dysmotility, sclerodactyly, and telangiectasia) syndrome was negative. She was initially started on prednisone followed by conventional disease-modifying antirheumatoid drugs with little benefit. She had a previous trial of anakinra (interleukin-1 [IL] antagonist) as well as canakinumab (IL-1 antagonist) with no clinical improvement.

**Figure 1.**
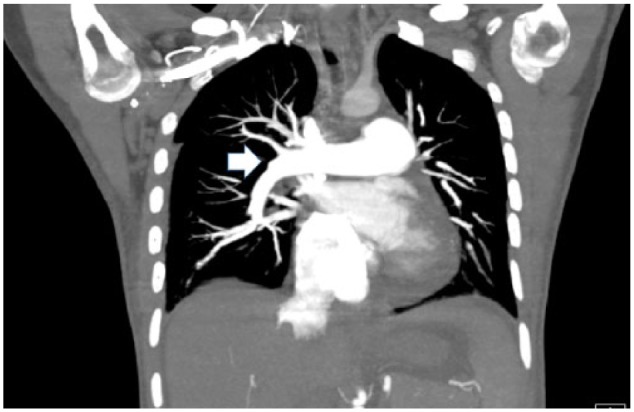
Computed tomography with contrast, coronal cut showing large pulmonary arterial trunk (arrow).

**Figure 2.**
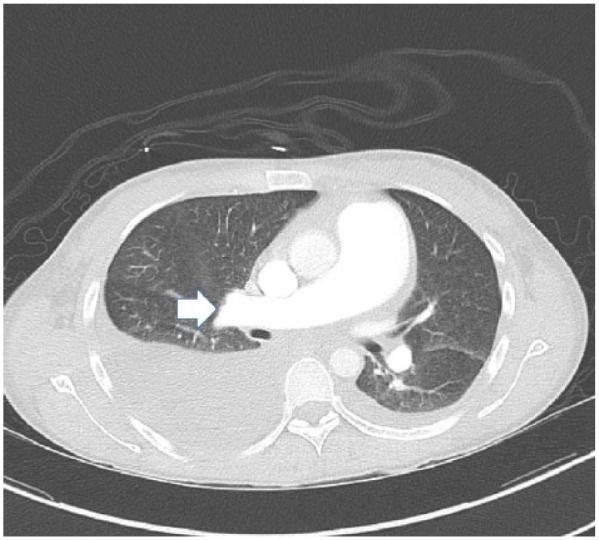
Computed tomography with contrast, axial cut showing markedly dilated pulmonary trunk (arrow).

On admission, posteroanterior radiograph of the chest showed an enlarged cardiac silhouette with bilateral pleural effusions. Echocardiogram showed severely dilated pulmonary artery with dilated right ventricle. The estimated right ventricular systolic pressure was markedly elevated to 74.64 mm Hg, with moderately reduced right ventricular systolic function. Left ventricular systolic function was normal with an ejection fraction of 56% to 60%. The patient also had bilateral pleural effusion for which chest tubes were placed. Computed tomography with contrast ruled out pulmonary embolism, but the findings were significant for severely dilated pulmonary arterial trunk (Figures 1 and 2). Rheumatoid factor and antinuclear antibody were negative. Anti-dsDNA antibody was negative. The patient had evidence of active inflammation; serum ferritin was very high: 1167.5 ng/mL (normal 11-306 ng/mL in females). Complement levels were obtained, C3 level was low at 65 mg/dL (normal 80-180 mg/dL) and C4 level was normal at 11 mg/dL (normal 10-45). Erythrocyte sedimentation rate was 9 mm/h, and C-reactive protein was 1.43 mg/L.

The patient was diagnosed with probable PAH with evidence of right heart failure. Right heart catheterization was considered, but could not be performed because of severely dilated pulmonary artery. Chest tubes were removed and the patient was transferred to a tertiary care center equipped to provide cardiopulmonary transplant.

## Discussion

AOSD is a clinical diagnosis, and an exclusion of other systemic disorders has to be carried out. The triad of fever, rash, and arthritis (Still’s triad) can be associated with macrophage activation syndrome, disseminated intravascular coagulation, thrombotic thrombocytopenic purpura, and diffuse alveolar hemorrhage.^1^ The Yamaguchi criteria for diagnosis of AOSD^2^ are summarized in Table 1. Apart from these criteria, elevated ferritin levels can also aid in the diagnosis of AOSD.^3^

**Table 1.** Yamaguchi Criteria for Diagnosis of AOSD.

Major criteria	• Fever >39°C, lasting 1 week or longer
	• Arthralgia or arthritis, lasting 2 weeks or longer
	• Typical rash
	• Leukocytosis >10 000/mm with >80% polymorphonuclear cells
Minor criteria	• Sore throat
	• Recent development of significant lymphadenopathy
	• Hepatomegaly or splenomegaly
	• Abnormal liver function tests
	• Negative tests for antinuclear antibody and rheumatoid factor (IgM)
Exclusion criteria	• Infections
	• Malignancies
	• Other rheumatic diseases
Five or more criteria are required with 2 or more being major criteria for diagnosis of AOSD	

Abbreviations: AOSD, adult-onset Still’s disease; IgM, immunoglobulin M.

As high as 40% of patients with AOSD have evidence of cardiopulmonary manifestations. PAH has been documented with AOSD, but is exceedingly rare with only 9 cases reported in literature between 1990 and 2016.^4^ As per the updated clinical classification of pulmonary hypertension, PAH associated with connective tissue disorders are categorized as Group 1.^5^ The primary pathology is intimal proliferation and medial hypertrophy ultimately causing narrowing of the arteriolar lumen. The mechanism of damage in AOSD is not entirely known but the presence of inflammatory cells in the perivascular regions of the remodeled vessels favors an inflammatory pathology.^6^ AOSD involves an auto-inflammatory response, with dysregulation of several cytokines. These include IL-1, IL-6, IL-18, and tumor necrosis factor-α, and high levels of these cytokines have been observed in AOSD as well as idiopathic PAH.

Echocardiography can suggest PAH with estimation of pulmonary artery pressure as well as right ventricular systolic pressure. Computed tomography scan of the chest may be performed to rule out interstitial lung involvement, or pathology favoring the diagnosis of other systemic diseases. Definitive diagnosis of PAH remains right heart catheterization with measurement of pulmonary capillary wedge pressure.

Survival after onset of PAH in patients with connective tissue diseases is shorter in comparison to idiopathic PAH.^7^ There have been no randomized clinical trials comparing treatment modalities for PAH secondary to AOSD and other connective tissue disorders, thus treatment is in the same lines as idiopathic PAH.^8^ Systemic inflammation plays a key role in the disease process of AOSD causing PAH. This is evident in studies that compared the use of anti-inflammatory/immunosuppressive therapy in conjunction with vasodilator therapies, and showed a significantly better response rate in comparison to vasodilator therapy alone.^9^

Treatment of PAH is aimed at the primary cause of disease, but Group 1 PAH often needs advanced therapy. There are reports of clinical improvement of PAH with immunosuppressive therapy, and advanced therapy inclusive of endothelin receptor blockers, prostanoids, and phosphodiesterase-5 inhibitors have been associated with improvement in exercise tolerance and pulmonary hemodynamics.^1^ IL-1 receptor antagonists (anakinra) has been reported to cause rapid regression of disease.^10^ IL-6 inhibitor use (tocilizumab) has been proposed for AOSD based on the disease pathology. Suggested treatment modalities based on our literature review have been summarized in Table 2.

**Table 2.** Suggested Treatment Modalities for PAH Due to AOSD Group 1 PAH.

Drug Group	Mechanism of Action	Example
Primary therapy		
Oral steroids	Immunosuppressive	• Prednisone
DMARD	Immunosuppressive	• Methotrexate
		• Cyclosporine
Drugs aimed at pulmonary hemodynamics		
Calcium channel blockers	Vasodilation, improved 5-year survival	• Dihydropyridine
		• Diltiazem
Prostaglandin analogs	Vasodilation	• Epoprostenol (continuous IV)
		• Treprostinil (IV and subcutaneous)
		• Selexipab (oral)
Endothelin antagonists	Vasodilation—as endothelin is vasoconstrictive and a mitogen	• Bosentan (nonselective)
		• Ambisentan (selective)
Nitric oxide–cyclic guanosine monophosphate enhancers	Vasodilation	• Sildenafil
		• Tadafil
Combination Therapy: May be needed for severe PAH. For Example, tadafil and ambisentan		
Biological agents aimed at AOSD		
Interleukin-1 antagonist	Cytokine blockade	• Anakinra
		• Canakinumab
Interleukin-6 antagonist	Cytokine blockade	• Tocilizumab

Abbreviations: PAH, pulmonary arterial hypertension; AOSD, adult-onset Still’s disease; DMARD, disease-modifying antirheumatic drug; IV, intravenous.

## Learning Points

- AOSD has been associated with PAH on rare occasions.
- Physicians should keep a vigil watch for respiratory symptoms like shortness of breath, cough, and pleuritic chest pain in a patient with AOSD.
- Specific therapy like anakinra and canakinumab should be attempted in addition to steroids.
- Early referral to higher centers equipped in dealing with pulmonary hypertension, heart failure, and possible cardiopulmonary transplant can be lifesaving.
